# Evaluation of cell-surface displayed synthetic consensus dengue EDIII cells as a potent oral vaccine candidate

**DOI:** 10.1186/s12934-018-0994-8

**Published:** 2018-09-14

**Authors:** Jyotiranjan Bal, Hee-Young Jung, Luong Ngoc Nguyen, Jisang Park, Yong-Suk Jang, Dae-Hyuk Kim

**Affiliations:** 1 Institute for Molecular Biology and Genetics, Department of Molecular Biology, Department of Bioactive Material Sciences, Chonbuk National University, Jeonju, Jeollabuk-do 54896 Republic of Korea; 2 Department of Biology, College of Sciences, Hue University, Hue, Vietnam

**Keywords:** Dengue, Oral vaccine, scEDIII, *Saccharomyces cerevisiae*, Surface display, Mucosal immunity

## Abstract

**Background:**

Dengue is a rapidly spreading mosquito borne tropical viral disease affecting hundreds of millions of people across the globe annually. The dengue virus (DENV) includes four genetically distinct serotypes that cause serious life-threatening infections, including dengue hemorrhagic fever/dengue shock syndrome. Dengue vaccine development is complicated by the possibility of vaccine-enhanced severe dengue disease due to antibody-dependent enhancement by pre-existing cross-reactivity, as well as homotypic antibodies. Thus, the development of an efficacious dengue vaccine conferring simultaneous and durable immunity to each of the four DENV serotypes has not yet been developed despite years of research. For mass immunization in deeply affected resource-limited countries, oral vaccination is considered more beneficial than conventional approaches. Therefore, in a continuing effort towards designing economical and potent vaccine candidates, the current study applied yeast surface display technology to develop an oral dengue vaccine candidate using whole recombinant yeast cells displaying the recombinant fusion protein of M cell targeting ligand Co1 fused to the synthetic consensus dengue envelope domain III (scEDIII). Female Balb/c mice were orally fed with recombinant yeast cells and immunogenicity in terms of systemic and mucosal immune responses was monitored.

**Results:**

Immunofluorescence microscopy with dengue specific antibody and fluorescein isothiocyanate-conjugated anti-mouse IgG antibody clearly showed that recombinant protein Co1-scEDIII-AGA was localized on the cell surface of the respective clones in comparison with scEDIII-Co1 and Mock cells with no fluorescence. Oral dosage applications of surface displayed Co1-scEDIII-AGA stimulated a systemic humoral immune response in the form of dengue-specific serum IgG, as well as a mucosal immune response in the form of secretory immunoglobulin A (sIgA). Antigen-specific B cell responses in isolated lymphoid cells from the spleen and Peyer’s patches further supported an elevated mucosal immune response. In addition, surface displayed Co1-scEDIII-AGA feeding elicited strong immune responses in comparison with scEDIII-Co1 and Mock following intraperitoneal booster with purified scEDIII antigen.

**Conclusions:**

Surface displayed preparations of Co1-scEDIII-AGA induced strong immunogenicity compared with non-displayed scEDIII-Co1. Prior studies have supported the neutralization potential of scEDIII constructs against all four serotypes. Thus, the oral administration of genetically engineered yeast whole cells displaying biologically active Co1-scEDIII fusion protein without any further processing shows prospective as a potent oral vaccine candidate against dengue viral infection.

## Background

Dengue, a mosquito-borne viral disease transmitted by female mosquitoes, in particular *Aedes aegypti* but also *A*. *albopictus* [1], affects an estimated 50–400 million people annually [2, 3]. Although considered to be a tropically neglected disease because of its major occurrence in the tropics, approximately 40% of the world’s population is at risk of its transmission. Apart from the increasing number of dengue-affected cases, explosive outbreaks also occur in newer regions, making it a major public health concern. Various factors, including the epidemiology of the four DENV serotypes and the complexity of immunoprotective and immunopathogenic responses following natural infection or vaccination, have created obstacles in the path of dengue vaccine development, despite 70 years of research [4]. Although the first licensed dengue vaccine CYD-TDV [5] has recently been approved, it was associated with an elevated risk of hospitalization for dengue among children younger than 9 years of age [6], and exhibited limited overall vaccine efficacy of 54% and reduced efficacy of 34% against DENV2, which is known to cause severe dengue infection and dengue outbreaks [7]. Thus, further studies on the development of potent and effective vaccines are required.

Dengue viruses (DENVs), the etiological agents of dengue disease, are positive-sense RNA viruses belonging to the family *Flaviviridae*. Comprised of four closely related, but antigenically distinct, serotypes (DENV-1, -2, -3, and -4), DENVs have major implications in severe disease consequences, vaccine-induced protection, and epidemic immensity [8]. Dengue infections are categorized as causing asymptomatic fever, dengue fever, and dengue hemorrhagic fever. During secondary infection, antibody-dependent enhancement (ADE) is responsible for the severe manifestations of dengue disease [9]. Therefore, an affordable vaccine with balanced and lasting immunity against all four DENV serotypes is required to prevent dengue infections.

The DENV open reading frame encodes a polyprotein that, upon post-translational processing, results in three structural proteins (capsid [C], membrane [M], and envelope [E]) and seven non-structural (NS) proteins (NS1, NS2A, NS2B, NS3, NS4A, NS4B, and NS5) [10], among which, the DENV E protein possesses immunomodulatory potential and assists in DENV entry, attachment, and serotype-specific antibody responses in the infected host [11, 12]. The EDIII domain of the DENV E protein, an immunoglobulin (Ig)-like C terminal domain [13, 14], consists of multiple potent and type-specific neutralizing epitopes [15] and functions as an effective antigen to elicit neutralizing antibodies in experimental animal models and thus represents a novel target for recombinant vaccine development [16]. To address the genetically distinct dengue serotypes, a consensus EDIII (cEDIII) immunogen was created through alignment of sequences from different isolates of the four serotypes of dengue virus, which elicits cross-neutralizing antibodies to block infections by each of the DENV serotypes simultaneously [17]. Yeast expressing scEDIII induced balanced immune responses against all four serotypes upon subcutaneous immunization in mice using purified protein emulsified in complete Freund’s adjuvant [18]. *Escherichia coli* heat-labile enterotoxin (LTB)-conjugated scEDIII produced neutralizing antibodies that elicited both humoral and cell-mediated immune responses [19].

Conventional vaccines are less economical and unsafe, and are therefore unsuitable for mass immunization in resource-limited countries. Oral vaccines have proven to be the best alternative as they avoid the risks commonly associated with conventional vaccines, and confer enhanced mucosal immune responses [20] and systemic responses, as well as being suitable for mass immunizations at a relatively low cost. In general, oral vaccines cause less stress and associated immune suppression for the recipient. Therefore, oral delivery is considered an ideal and easy route to introduce foreign antigens.

Mucosal surfaces play an important role in providing the first line of defense against pathogens. Antigen targeting to the mucosal tissue is essential for effective oral tolerance and initiation of active immune responses. Vaccines delivered into the mucosal immune system can induce effective systemic immune responses simultaneously with mucosal immunity in a manner that is comparable to conventional vaccination [21]. Diverse molecules employed to target vaccine antigens to mucosal and systemic compartments have been characterized. In particular, M cells, the specialized epithelial cells of mucosa-associated lymphoid tissue, are responsible for antigen uptake and their rapid and effective transcytotic activity makes them an attractive target for mucosal vaccine delivery [22]. M cell-specific targeting of the tetravalent dengue antigen (Tet-EDIII) via the Co1 ligand has been performed previously [23]. Co1-conjugated EDIII antigens are also known to be efficiently delivered into Peyer’s patches (PP), which facilitates the generation of mucosal immune responses [24]. Furthermore, antibodies induced by the ligand-conjugated EDIII antigen showed effective virus-neutralizing activity.

*Saccharomyces cerevisiae*, apart from being considered safe (GRAS), is a simple, low cost, and robust eukaryotic expression system with known tools of genetic manipulation that possesses inherent advantages of eukaryotic post-translational modifications and secretion, and the cells are suitable to be taken up by antigen-presenting cells (APCs) [25]. Furthermore, from the vaccine point of view, recombinant *S*. *cerevisiae* are potent transporters of target protein and expression vector DNA into dendritic cells, triggering antigen-specific CD4+ and CD8+ immune responses in vivo [26, 27]. Oral immunization of recombinant *S*. *cerevisiae* activates potent innate, as well as adaptive, T cell immune responses to the target antigen [19, 28]. The above properties make *S*. *cerevisiae* a potent nonpathogenic vaccine-delivery vehicle.

Yeast surface display technology represents an efficient approach for antigen presentation in the cell. It is commonly used for functionalizing yeast cells for a variety of biotechnological applications, including generation of whole-cell biocatalysts, antimicrobial agents, and oral vaccines [29]. Integration of the protein of interest to the C- or N-terminus of an anchor protein on the cell surface of *S*. *cerevisiae* typically results in the display of up to 100,000 copies of the fusion protein [30]. Several studies have proposed the suitability of antigen-displaying yeast for preventative or therapeutic oral vaccines [31, 32]. *Pichia pastoris* surface displayed hemagglutinin protein from a highly pathogenic avian influenza virus, subtype H5N1, and produced virus neutralizing antibodies upon oral administration [33]. A neutralizing epitope of ApxIIA exotoxin of Korean *Actinobacillus pleuropneumoniae* displayed on the *S*. *cerevisiae* cell surface elicited strong immune responses when administered orally and was sufficient to protect against pathogen infection [34]. Oral delivery of *S*. *cerevisiae* cells displaying Eno1p on their cell surfaces protected 60% of mice against candidiasis [35]. The yeast surface display system is also advantageous as it provides antigenic proteins more rapidly and conveniently than conventional approaches of vaccine production.

Taking into consideration the immunogenic efficacy of scEDIII, the efficient mucosal targeting, elicitation of immune responses by Co1, and advantages of the surface display technology of *S*. *cerevisiae*, we evaluated the immunogenicity of the fused recombinant antigen surface displayed preparations of *S*. *cerevisiae* as an oral dengue vaccine candidate in a female BALB/c mouse model.

## Methods

### Animal housing and ethics statement

Female BALB/c mice, aged 5 weeks, were procured from the Charles River Laboratories through Orient Bio, Inc. (Sungnam, Korea) and maintained under general specific pathogen-free conditions. Six mice per filtertop microisolator cage were housed in a temperature- and humidity-controlled room. Mice were acclimated for approximately 1 week prior to initiation of the oral feeding experiment. Experimental procedures involving laboratory animals strictly adhered to the guidelines established by the Institutional Animal Care and Use Committee of the Chonbuk National University (Approval Number: CBU 2015-0004).

### Reagents

An anti-dengue virus primary monoclonal antibody (mAb) was procured from LifeSpan BioSciences, Inc. (Seattle, WA, USA). This antibody recognizes all four dengue virus serotypes (DENV-1, DENV-2, DENV-3 and DENV-4) of the genus Flavivirus. An anti-mouse IgG (whole molecule)-fluorescein isothiocyanate (FITC) antibody produced in goat was purchased from Sigma-Aldrich (St. Louis, MO, USA).

### Construction of fusion protein expression vectors

**Fig. 1:**
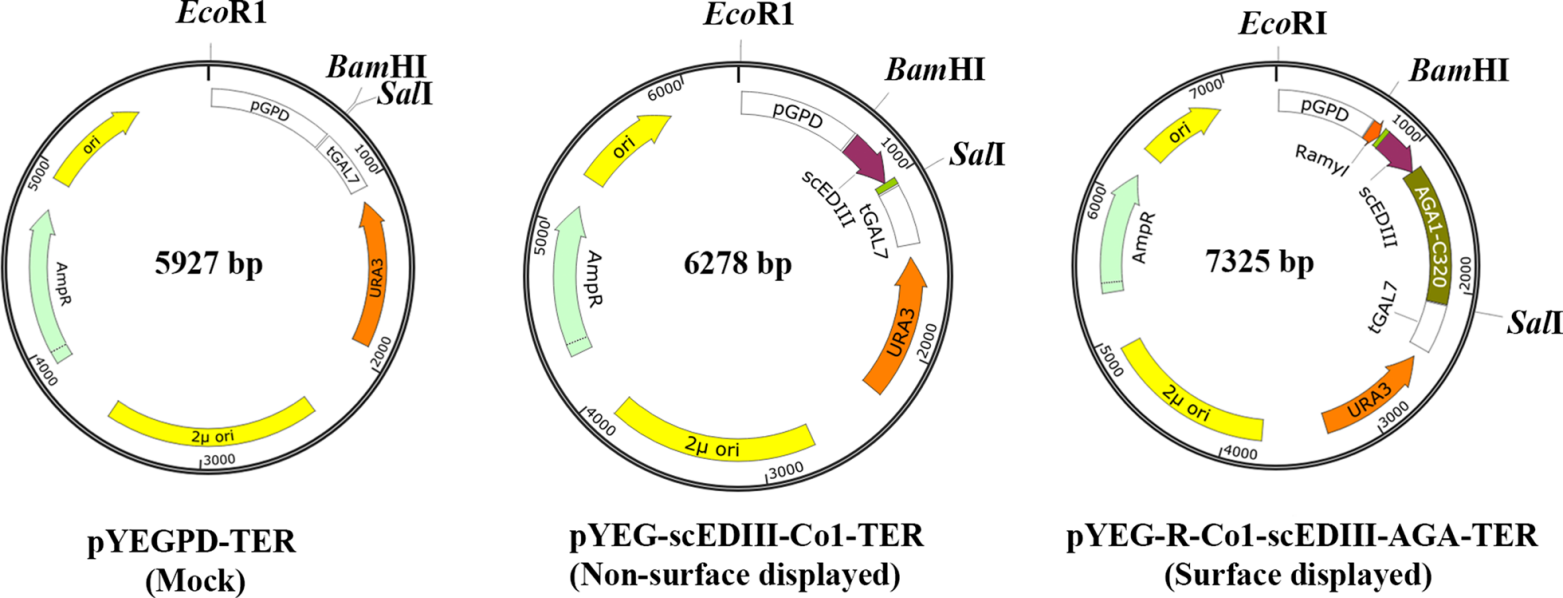
Expression vector design. Schematic illustration of the expression plasmids pYEGPD-TER (Mock), pYEG-scEDIII-Co1-TER (non-surface displayed), and pYEG-R-Co1-scEDIII-AGA-TER (surface displayed)

To construct the expression vector for surface display of the recombinant protein scEDIII, the amylase 1A (Ramy1A) signal peptide, the M-cell specific ligand (Co1) gene, target gene fragment scEDIII, and an anchor DNA fragment containing the 3′ half of the α-agglutinin gene (AGA1-C320) encoding the C-terminal 320 amino acids [36] were fused sequentially by overlap extension polymerase chain reaction (PCR) using primers listed in Table 1 and DNA polymerase from Takara Bio Inc. (Otsu, Japan). *S*. *cerevisiae* codon-optimized scEDIII gene [18] was amplified from pUC19 harboring scEDIII. Furthermore, for the construction of the control strain lacking surface display of the scEDIII antigen, the Co1 gene and scEDIII alone were fused through overlap extension PCR using the primers listed in Table 1. In addition, *Bam*HI and *Sal*I restriction sites were included at the 5′ and 3′ ends of both the fusion constructs, respectively, to facilitate subsequent cloning. The fusion constructs were cloned into the yeast episomal shuttle vector, pYEGPD-TER, to construct pYEG-R-Co1-scEDIII-AGA-TER with glyceraldehyde-3-phosphate dehydrogenase (GPD) as the promoter and galactose-1-phosphate uridyl transferase (GAL7) as the terminator (Fig. 1). The resulting clones were confirmed through restriction enzyme digestion and DNA sequencing. The expression host *S*. *cerevisiae* strain 2805 (*MATα pep4::HIS3 prb1*-Δ*can1 GAL2 his3 ura3*-*52*) [37] was transformed by pYEG-R-Co1-scEDIII-AGA-TER and pYEG-scEDIII-Co1-TER using the lithium acetate method [38]. Empty vector pYEGPD-TER-transformed yeast (termed Mock) was used as a negative control.

**Table 1:** Oligonucleotide primers used in this study

Primers	Sequence (5′–3′)^a^
Co1-scEDIII-AGA construction	
Ramyl-F	GGATCCGCATGCAGGTGC
Ramyl-Co1 OR	TAGCTGGTAGTTGATGGAAAGACCCGGCTGTCAAGT
Co1-scEDIII F	TCAACTACCAGCTAGAAGTCCACTACCAAAAGGAATGTCT
scEDIII-AGA OF	TAAAAAGGGTTCCTCAGCCAAAAGCTCT
scEDIII-AGA OR	AGAGCTTTTGGCTGAGGAACCCTTTTTA
AGA-R	GTCGACGCTTAGAATAGCAGGTACGACAA
scEDIII-Co1 construction	
scEDIII-Co1-F	CGGGATCCCGATGAAAGGAATGTCTTACGCA
scEDIII-Co1-R	GTCGACCTATGGTAGTGGACTTCTAGCTGGTAG TTGATGGAAAGATGAGGAACCCT

^a^Underlined nucleotides indicate engineered restriction sites used in cloning

### Northern blot analysis to confirm expression of the target gene and selection of efficient clones

As described previously [18], total yeast RNA was extracted from the selected clones, separated on a 1.2% formaldehyde-agarose gel, transferred to an Amersham Hybond™ membrane, and hybridized with an α-[^32^P]-labeled probe generated using a random labeling kit (Amersham Pharmacia Biotech, Piscataway, NJ, USA). The developed blot visualized the expression pattern of scEDIII-Co1 and Co1-scEDIII-AGA along with *S*. *cerevisiae* GPD as a reference.

### Immunostaining and fluorescence microscopy

Immunofluorescence labeling of recombinant yeast cells was performed to confirm the expression and surface display of Co1-scEDIII-AGA through a slightly modified method, as described previously [36, 39]. Cultivated recombinant yeast cells (48 h) were washed three times with phosphate-buffered saline (PBS). The pellet was resuspended in PBS containing 1% bovine serum albumin (BSA) and incubated for 30 min at room temperature. Cells were then washed three times with PBS, resuspended in PBS containing 3% BSA and polyclonal anti-dengue mouse IgG (1:100), and incubated at room temperature for 2 h. Next, cells were washed as described above, the pellet was resuspended in PBS containing 3% BSA and secondary antibody, FITC-conjugated goat anti-mouse IgG (1:50; Sigma-Aldrich), and incubated at room temperature for 1 h. After washing, the cells were observed using confocal laser scanning microscopy (Carl Zeiss, Zena, Germany).

### Culture conditions and target protein expression analysis

The *S*. *cerevisiae* transformants containing pYEG-R-Co1-scEDIII-AGA-TER and pYEG- scEDIII-Co1-TER were cultured and maintained in uracil-deficient (ura^−^) selective medium, as described previously [19]. The *E*. *coli* strains were maintained in Luria–Bertani broth with the appropriate antibiotics.

For target protein expression in non-surface displayed yeasts, cell-free extracts (CFEs) were prepared, as described previously [18]. The cell wall fraction (CWF) was extracted and analyzed to confirm expression of the target protein at the cell surface. Cultured cells were resuspended in 200 µL of extraction buffer (50 mM Tris–Cl, pH 8.0; 2% SDS; 10 mM DTT; 0.1 M EDTA) and boiled for 30 min, followed by centrifugation at top speed for 20 min at 4 °C. After suspension, samples were boiled for 30 min and centrifuged at top speed for 20 min at 4 °C. After centrifuging, the supernatant was transferred into a 1.5 mL EP tube. Protein concentration was determined using the Bio-Rad Protein Assay Kit (Bio-Rad, Hercules, CA, USA). Sample aliquots of the CFEs and CWF were separated by SDS–polyacrylamide gel electrophoresis and transferred onto Hybond-C Extra nitrocellulose filter membranes (Hybond, Amersham Pharmacia Biotech). Primary anti-dengue virus mAb (LifeSpan BioSciences, Inc.) that recognized scEDIII was used to detect target proteins. After incubation with goat anti-mouse IgG alkaline phosphatase conjugate (Sigma-Aldrich), the blots were developed by BCIP/NBT in TMN buffer (100 mM Tris, pH 9.5; 5 mM MgCl_2_; and 100 mM NaCl). Purified protein extract from the BL21 *E*. *coli* strain expressing scEDIII [16] was used as a positive control.

### Preparation of oral doses for vaccination

In accordance with the successful elicitation of an immune response due to the oral dose of 1.6 g fresh weight of recombinant yeast cells in our previous study [19], the same dose was used for the oral vaccination of mice. Briefly, ~ 20 g fresh weight of recombinant yeast cells was harvested from 640 mL of culture and subsequently divided into aliquots containing 1.6 g of recombinant yeast cells for a single dose.

### Oral immunization and blood and fecal sampling

**Fig. 2:**
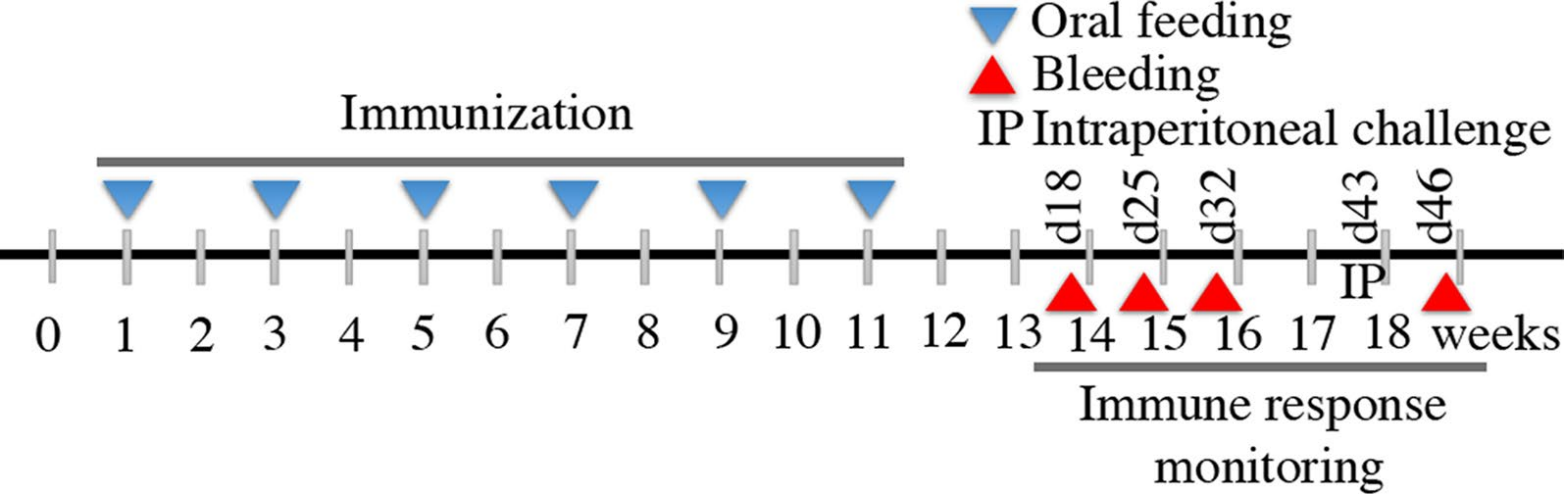
Schematic representation of the oral immunization protocol. Each mouse in a group of six was orally gavaged with 1.6 g fresh weight of cells resuspended in a final volume of 2.4 mL of phosphate-buffered saline. A total of six doses were orally administered every alternate week with each dose consisting of cell suspensions that were split equally and administered four times per day. Subsequent immune response monitoring was performed through retro-orbital bleeding and fecal matter collection at 4 days after the final feeding and continued every week up to 32 days, followed by intraperitoneal booster injection with purified antigen on day 43 and subsequent bleeding on day 46. d# represents days post final vaccination

The oral immunization strategy and schedule were followed as described previously with minor modifications [19]. Briefly, mice groups were fasted overnight (water was provided ad libitum). Oral feeding with whole recombinant yeast cell preparations was performed using a 1-mL syringe fitted with an oral feeding needle. The three groups consisting of six BALB/c mice were fed separately with Mock, Co1-scEDIII-AGA, or scEDIII-Co1. Each BALB/c mouse was administered a total of six doses, with each dose consisting of 1.6 g fresh weight of cells, resuspended in a final volume of 2.4 mL of PBS, split equally, and orally gavaged four times every alternate week, as depicted in Fig. 2 with minimum distress. To investigate the memory response, an intraperitoneal injection of 20 µg of alum-adsorbed purified *E*. *coli*-expressed scEDIII was administered to each immunized mouse 3 weeks after the last oral immunization. Blood and fecal pellets were collected periodically from each mouse at day (d) 18, d25, and d32 after the last oral immunization (Fig. 2). Blood was collected through retro-orbital bleeding, maintained at room temperature for 1 h to clot, and kept overnight at 4 °C to facilitate clot retraction before serum was recovered for storage at −20 °C. Extracts from the collected fecal matter were prepared in PBS for enzyme-linked immunosorbent assay (ELISA) analysis. To observe memory responses, weeks after the final immunization, intraperitoneal injection of a booster dose of *E*. *coli* expressed purified scEDIII antigen was administered. Retro-orbital bleeding was then performed after 3 days of injection.

### ELISA detection of anti-dengue antibodies

Changes in humoral immune responses upon oral administration of recombinant yeast cells were analyzed by estimating the antibodies induced through indirect ELISAs using NUNC Maxisorp 96-well ELISA plates coated with 100 ng/well of *E*. *coli*-expressed recombinant scEDIII protein. The plates were washed three times with PBS + 0.05% Tween 20 and blocked with 1% BSA in PBS for 2 h at 37 °C. Following the washes, twofold serial dilutions were performed after adding 100 μL/well of sera from immunized mice (the starting dilution points were 1:25 for serum IgG and 1:2 for fecal sIgA), and the plates were incubated overnight at 4 °C. Alkaline phosphatase (AP)-conjugated secondary antibodies (anti-mouse IgG or IgA; Sigma-Aldrich) diluted 1:5000 in PBS containing 0.5% BSA were added and incubated for 2 h at 37 °C followed by a washing step. To detect the response, 100 μL/well of phosphatase substrate (S0942, Sigma-Aldrich) was added and incubated for 15 min at room temperature. The reaction was stopped with 2 M H_2_SO_4_ and the optical density was measured at 405 nm using a microplate reader (Multiskan™ GO Microplate Spectrophotometer, Thermo Fisher Scientific Inc., Waltham, MA, USA).

### Enzyme-linked immunosorbent spot (ELISPOT) assay

ELISPOT assay to enumerate IgG or IgA secreting cells in lymphocytes isolated from the spleen and PPs of immunized mice was conducted as described previously [40, 41]. The PPs were carefully excised from the small intestines of two mice randomly selected from a group of six on d39 before booster immunization and further dissociated into single T cells by stirring with collagenase D (0.5 mg/mL) and DNase I (100 μg/mL) for 60 min at 37 °C.

### Statistical analysis

The statistical significance of the difference between the experimental parameters was determined using the two-tailed Student’s *t*-test and analysis of variance, wherever indicated, using GraphPad Prism Mac version 6.0e. *p*-values < 0.05 were considered to indicate significance.

## Results

### Creation of an *S*. *cerevisiae* strain displaying scEDIII on its cell surface

To create an *S*. *cerevisiae* strain displaying recombinant scEDIII protein on its cell surface, the plasmid pYEG-R-Co1-scEDIII-AGA-TER was constructed, as described in “Methods” (Fig. 1). This expression plasmid is a multicopy plasmid for expression of the fusion gene containing the secretion signal sequence Ramy1A under control of the GPD promoter. The protein of interest is secreted due to the Ramy1A signal sequence and is anchored to the cell surface via AGA1. Following transformation of the above plasmid into *S*. *cerevisiae* strain 2805, more than 20 transformants were randomly selected on ura− medium and were further examined using colony PCR and *E*. *coli* back transformation to confirm the presence of the corresponding recombinant plasmid. To compare and analyze the beneficial effects due to the surface display of the target antigen, an *S*. *cerevisiae* strain expressing only the M cell targeting ligand Co1 conjugated to scEDIII and another expressing empty vector only, also referred to as Mock, respectively, were created as controls.

**Fig. 3:**
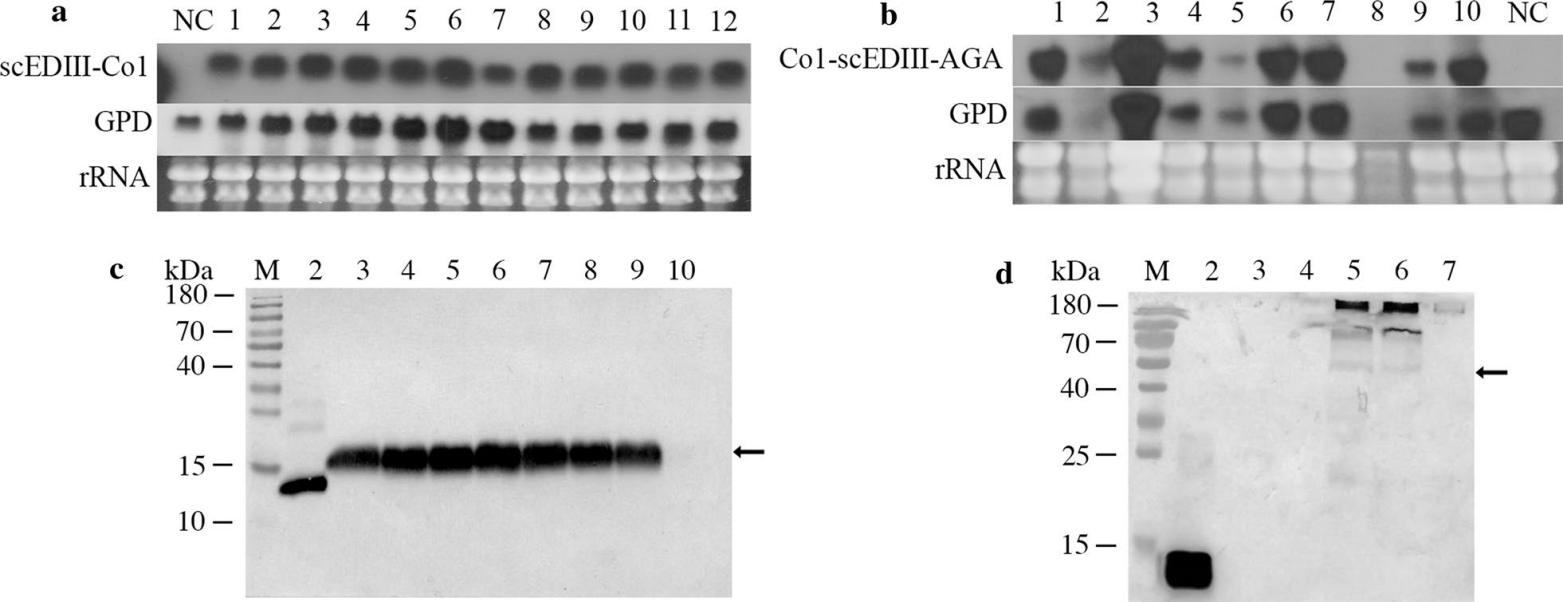
Expression analysis of recombinant proteins. **a** Northern blot analysis showing levels of Co1-scEDIII transcripts from selected transformants. Lane NC contains RNA from mock-transformed cells. Lanes 1–12 consist of RNA from the corresponding pYEG-Co1-scEDIII-TER transformants. Glyceraldehyde-3-phosphate dehydrogenase (GPD) transcription is shown as an internal control. Ribosomal RNA (rRNA) was used as a loading control. **b** Northern blot analysis showing levels of Co1-scEDIII-AGA transcripts from selected transformants. Lanes 1–10 consist of RNA from the corresponding pYEG-Co1-scEDIII-AGA-TER transformants. Lane NC contains RNA from mock-transformed cells. GPD transcription is shown as an internal control and rRNA was used as a loading control. **c** Western blot analysis of recombinant protein expression using anti-dengue IgG antibodies (lanes 2–10). Lane marked as ‘M’ represents pre-stained protein size marker. Their sizes (in kDa) are indicated to the left of the first lane. The arrow indicates the position of the recombinant scEDIII-Co1 protein. Lane 2 contains 1 µg of cell-free extract (CFE) from *Escherichia coli*-expressed scEDIII [14]. Lanes 3–9 contain 40 µg of CFE from recombinant Co1-scEDIII strains. Lane 10 contains 40 µg of CFE from Mock as negative controls. **d** Western blot analysis to confirm the expression of surface displayed recombinant Co1-scEDIII-AGA protein using anti-dengue IgG antibodies (lanes 2–7). Lane marked as ‘M’ represents pre-stained protein size marker. Their sizes (in kDa) are indicated to the left of the first lane. The arrow indicates the position of the recombinant Co1-scEDIII-AGA protein. Lane 2 contains 1 µg of CFE from *E*. *coli*-expressed scEDIII. Lane 3 contain 40 µg of CFE from wild type *Saccharomyces cerevisiae* 2805 strain. Lane 4 contain 40 µg of CFE from the Mock control. Lanes 5 and 6 contain CFEs and cell wall fractions from recombinant Co1-scEDIII-AGA strains, respectively. Lane 7 contains 10 mL of culture filtrate extracted from Co1-scEDIII-AGA strains

Northern blot analysis of the selected transformants using fusion gene constructs revealed the accumulation of scEDIII transcripts in all transformants (Fig. 3a, b). Among the transformants harboring Co1-scEDIII-AGA, one was selected (#7 strain showing the highest transcript level), whereas among transformants harboring scEDIII-Co1, #8 based on the high expression level relative to the internal control (GPD) was selected for subsequent experiments.

### Western blot analysis to confirm the expression of recombinant scEDIII

Western blot analysis of CFEs of the strain harboring pYEG-Co1-scEDIII-TER showed a corresponding scEDIII-Co1 protein band at ~ 15 kDa confirming the expression of Co1-scEDIII (Fig. 3c). To further verify the expression of recombinant scEDIII and its association with the yeast cell wall, the selected *S*. *cerevisiae* transformant strain #7 harboring the pYEG-R-Co1-scEDIII-AGA-TER plasmid was cultured, followed by isolation of the CWF and subsequent extraction of cell wall proteins (CWPs). Isolation of the CWF from negative controls, such as Mock and wild-type cells, were also extracted. Western blot analysis to confirm the identity of the recombinant protein using anti-dengue antibody revealed that the CWPs of cells harboring pYEG-R-Co1-scEDIII-AGA-TER showed a band corresponding to the size of the fusion protein of Co1-scEDIII-AGA with the expected size of ~ 50 kDa (Fig. 3d), whereas no corresponding band was found in the culture filtrate of the transformant cells or in the cell fractions of Mock cells or wild-type cells, confirming the anchorage of the target protein Co1-scEDIII-AGA to the yeast cell wall.

### Fluorescence labeling for detection of the surface displayed recombinant protein Co1-scEDIII-AGA

**Fig. 4:**
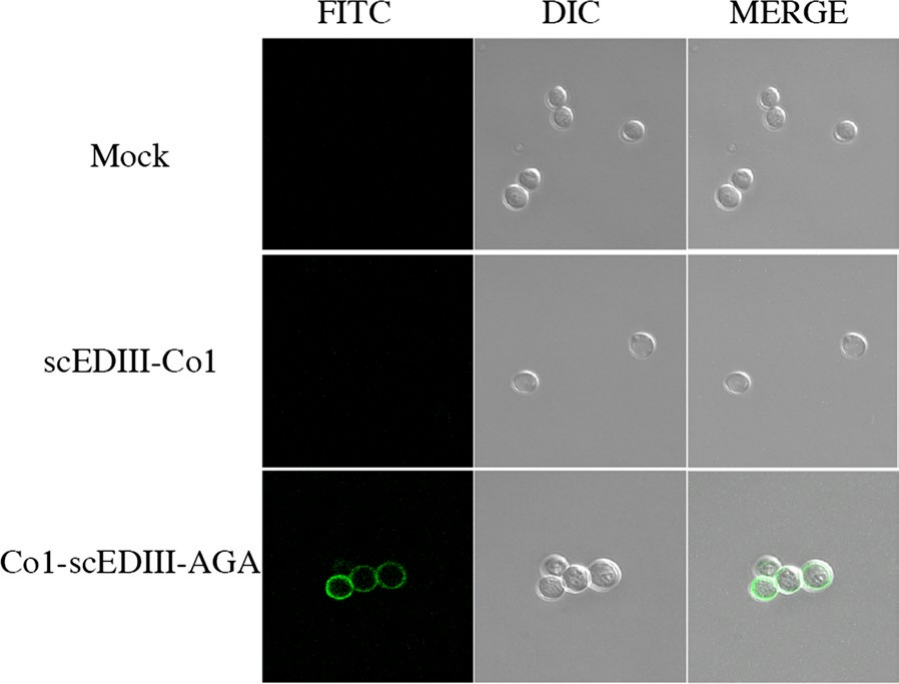
Immunofluorescence labeling of recombinant yeast cells. Immunofluorescence microscopic observation of displayed Co1-scEDIII-AGA on *S*. *cerevisiae*. Fluorescence microscopic images after staining recombinant yeast cells using the anti-dengue mAb followed by fluorescein isothiocyanate-conjugated anti-mouse IgG. The surface displayed protein is visualized as a ring on the cell wall of yeast cells

To confirm the secretion and targeting to the cell surface of the expressed Co1-scEDIII-AGA, recombinant yeast cells in the exponential growth phase were labeled with FITC-conjugated antibodies followed by visualization using confocal laser scanning microscopy (Fig. 4). The cell walls of recombinant strains harboring pYEG-R-Co1-scEDIII-AGA-TER fluoresced and were visualized as a ring, in contrast to no fluorescence in the mock transformant and the strain harboring pYEG-scEDIII-Co1-TER. This confirms the specificity of the immunofluorescence labeling and correct localization of the Co1-scEDIII-AGA antigen on the yeast cell surface. The absence of fluorescence in other parts of the cell indicates no cross-reactivity of dengue antibody with recombinant yeast cells.

### Surface displayed Co1-scEDIII elicits a systemic humoral immune response upon oral immunization

**Fig. 5:**
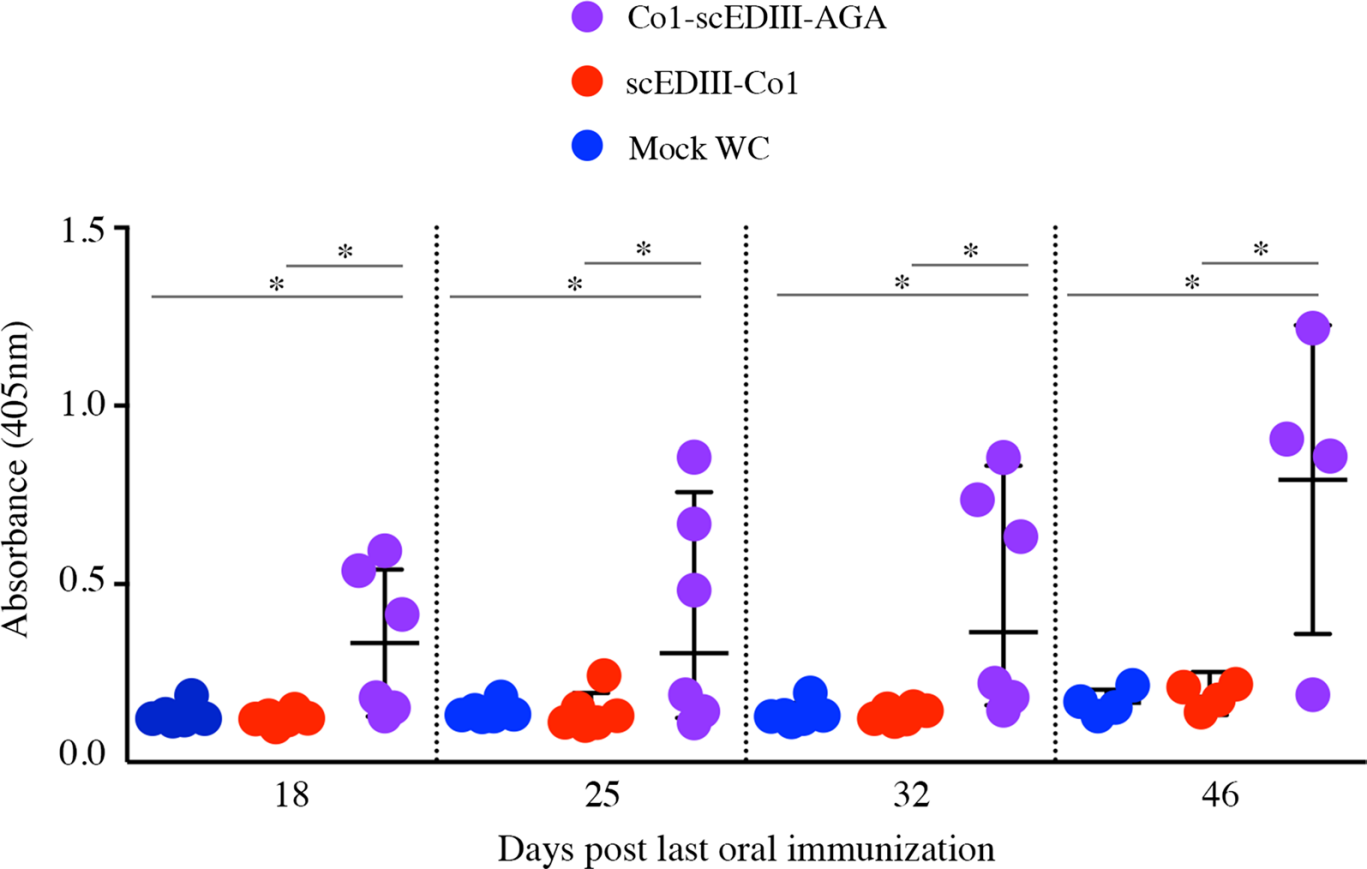
Humoral IgG responses in orally immunized mice. Dengue-specific serum IgG was induced in mice that received oral administration of scEDIII-Co1 and Co1-scEDIII-AGA, and this induction was observed at 4 days after the last immunization until day 32, as measured at weekly intervals. On day 46, following booster immunization with purified *E*. *coli* expressed scEDIII protein, dengue-specific serum IgG levels were also observed. An unpaired Student’s *t*-test was used to calculate *p*-values, and *p < 0.05 indicates significant differences between the groups

M-cell targeting Co1 ligand is an effective mucosal targeting molecule and *S*. *cerevisiae* cells have multiple adjuvant properties apart from being GRAS, thereby making them an ideal combination to elicit immune responses. Thus, we explored the immunogenicity of the surface displayed yeast that expressed Co1-scEDIII-AGA through oral administration. Mice immunized with Co1-scEDIII-AGA showed significantly higher scEDIII-specific IgG antibody titers compared to Mock, which showed no dengue specific IgG antibodies (Fig. 5). Furthermore, Co1-scEDIII-AGA immunized mice showed significantly higher dengue specific antibody titers compared to non-surface displayed scEDIII-Co1 immunized mice. However, mice immunized with scEDIII-Co1 showed no significant levels of dengue-specific IgG compared with those immunized with Mock. The detected immune responses showed prime-boost-dependent kinetics, with antibody titer sharply rising following the second boost.

### Oral immunization of surface displayed Co1-scEDIII elicited a mucosal immune response

**Fig. 6:**
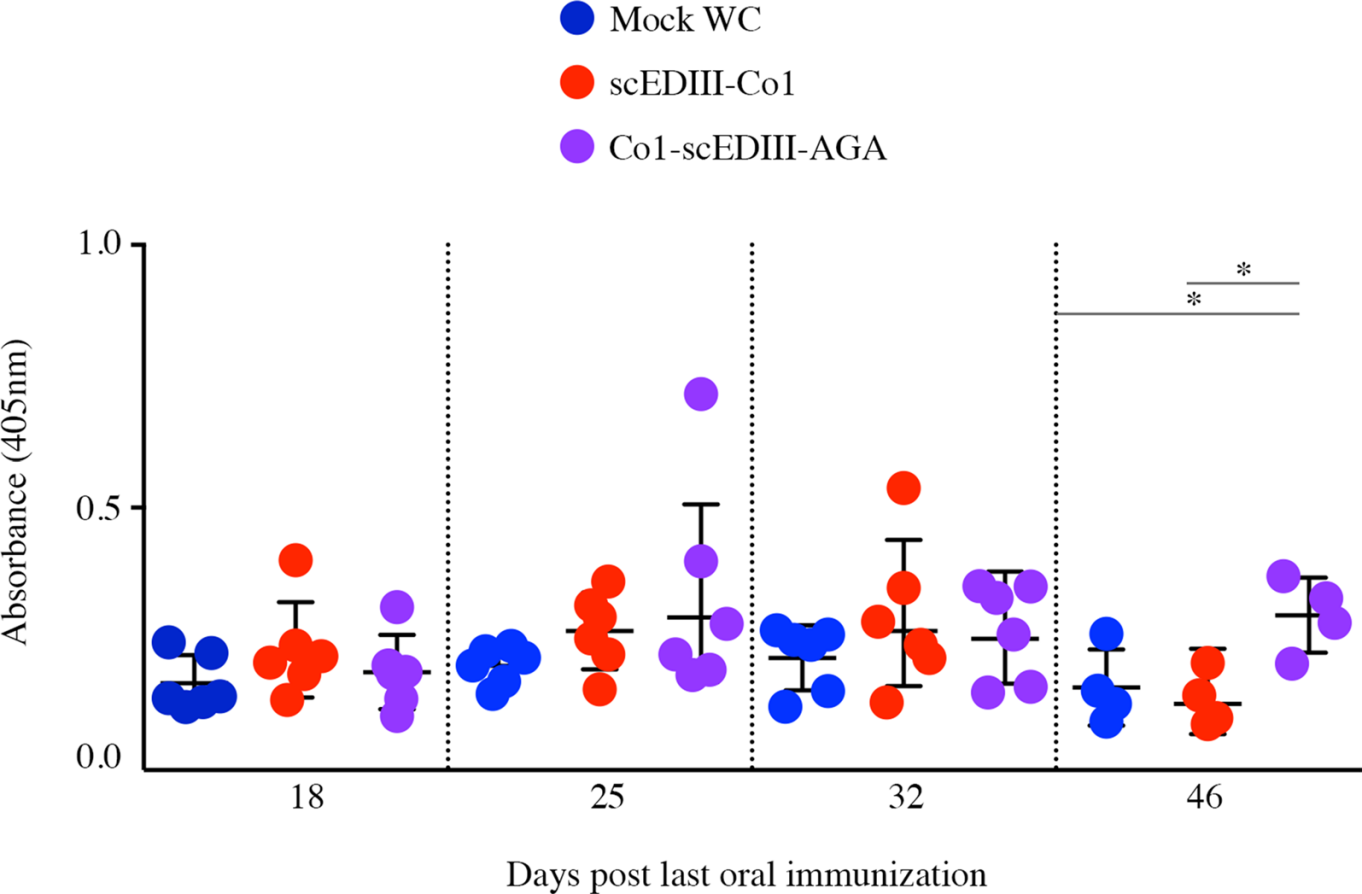
Mucosal sIgA responses upon oral immunization. scEDIII-specific fecal sIgA was induced in mice immunized orally with recombinant scEDIII-Co1 and Co1-scEDIII-AGA at 4 days after the last immunization until day 32, observed at weekly intervals. Dengue-specific fecal sIgA levels were also observed on day 46, 3 days after booster immunization with purified *E*. *coli* expressed scEDIII protein. An unpaired Student’s *t*-test was used to calculate *p*-values, and *p < 0.05 indicates significant differences between the groups

The effectiveness of an oral vaccine depends on activation of the mucosal immune response system. Therefore, the mucosal immune response in orally immunized mice was estimated through dengue specific fecal sIgA antibody titers with ELISA. Mice immunized with surface displayed Co1-scEDIII-AGA elicited scEDIII-specific fecal sIgA. However, the dengue specific sIgA levels for mice immunized with surface displayed Co1-scEDIII-AGA were higher on d25 post last oral immunization, but were not significantly different from mice immunized with scEDIII-Co1 or mock transformant (Fig. 6). These results suggest that scEDIII was targeted to the mucosal system via surface displayed cells, despite the lower mucosal immune response.

### ELISPOT assay enumerates the number of IgG- and sIgA-secreting cells upon oral immunization of surface displayed Co1-scEDIII

**Fig. 7:**
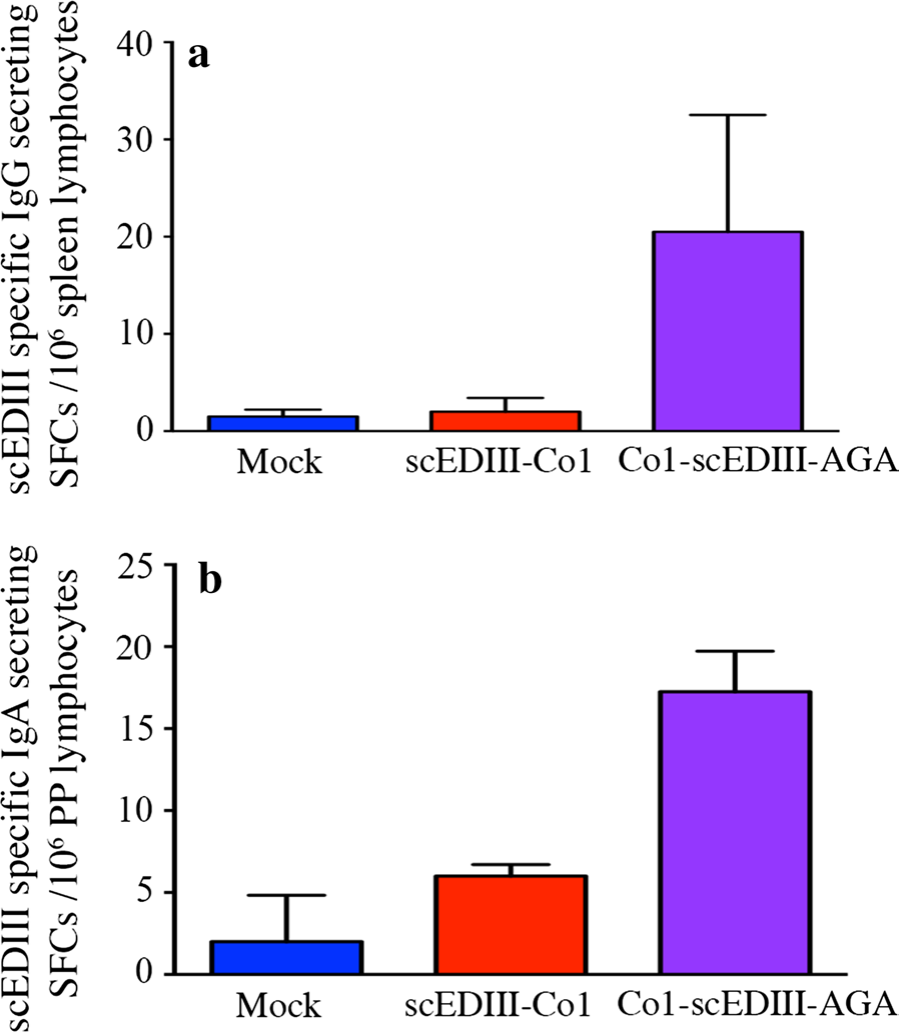
Frequency of antigen-specific immune cells in orally immunized mice. Enzyme-linked immunosorbent spot assay showing (**a**) scEDIII-specific IgG and (**b**) scEDIII-specific IgA antibody spot forming cells in lymphocytes isolated from spleens and Peyer’s patches of immunized mice, respectively. Each group represents two mice analyzed individually in triplicate. This is a representative result of two independent experiments showing similar results

Apart from heterogeneity, conventional ELISA revealed antibody titers that were below threshold levels. To confirm further the scEDIII-specific mucosal immune responses, we performed ELISPOT assays, which are much more sensitive than conventional ELISAs, on lymphocytes isolated from mucosal tissues such as the spleen and PPs from immunized mice. Briefly, on d39 before booster immunization, two randomly selected mice were sacrificed from each group, and their spleens and PPs were retrieved. Lymphocytes were isolated from the tissues and probed for scEDIII-specific IgG- and IgA-secreting cells using ELISPOT assays. The number of scEDIII-specific antibody forming cells (AFCs) in the form of spot-forming cells in the spleen and PP lymphocytes differed considerably between surface displayed Co1-scEDIII-AGA administered mice and their scEDIII-Co1 and Mock-administered counterparts (Fig. 7). Furthermore, comparatively higher numbers of scEDIII-specific IgG AFCs in splenic lymphocytes and scEDIII-specific IgA AFCs in the PP lymphocytes were observed in the surface displayed Co1-scEDIII-AGA compared to scEDIII-Co1 mice. The mucosal cells from Mock-fed mice showed no scEDIII-specific IgG AFCs or IgA AFCs in mucosal cells. These results suggest that the enhanced levels of scEDIII-specific serum IgG and fecal IgA were caused by efficient targeting to the mucosal immune system due to the display of the target antigen on yeast cell surface and presence of Co1 on the fusion protein.

### Oral immunization elicits an elevated immune response upon booster immunization

Long-term memory response upon antigenic challenge is an essential attribute of an efficient vaccine. To determine whether a memory response had been established as a result of oral immunization, we evaluated serum IgG levels following administration of an intraperitoneal booster injection of 20 µg of alum-adsorbed purified *E*. *coli*-expressed scEDIII to each immunized mouse on d43 following the last oral immunization. The secondary immune response due to oral immunization of surface displayed Co1-scEDIII-AGA was significantly higher compared to both Mock and scEDIII-Co1 upon intraperitoneal antigenic booster dose (Figs. 5, 6). Both IgG and sIgA titers with surface displayed Co1-scEDIII-AGA were significantly higher than those of the booster dose immunized Mock and scEDIII-Co1, respectively. These results suggest that a strong memory response may have been elicited upon oral immunization with surface displayed Co1-scEDIII-AGA in contrast to that of non-displayed scEDIII-Co1.

## Discussion

Dengue is considered a rapidly spreading fatal disease across the globe, and an efficacious and economical vaccine is required for mass immunization to prevent dissemination. In our previous studies on recombinant dengue vaccine development, we first confirmed targeted delivery of an M cell-specific Co1 ligand-conjugated recombinant tetravalent gene comprising the amino acid sequences of dengue envelope domain III (Tet-EDIII) from four serotypes into M cells in PPs [23]. Furthermore, yeast-expressed scEDIII possessed a balanced immune response against all four serotypes [18]. Subsequently, we evaluated *S*. *cerevisiae*-expressed *E*. *coli* LTB-conjugated scEDIII as an oral vaccine candidate in the form of whole cells (WCs) or CFEs, both of which elicited mucosal as well as cell-mediated immune responses, with the exception that the immune response due to WCs was lower than that of CFEs [19]. Earlier observations showed that WCs have an additional advantage compared to CFEs in the form of oral vaccines. Soluble antigens do not sufficiently penetrate the mucus layer of the intestine. As a result, these antigens are taken up marginally by APCs and generate tolerance [42]. Immune responses against the wild-type yeast cell were shown to be higher than that of the hepatitis B surface antigen protein HBS3 [43]. Therefore, considering the advantages of WCs, an attempt was made in the present study to further improve the efficacy of the WC formulation using a combination of two strategies, one of which includes yeast surface display technology, which presents the antigenic proteins more rapidly and conveniently, and the second of which includes the use of mucosal M cell targeting ligand Co1 for efficient mucosal targeting.

The EDIII domain of the DENV E protein is a known effective antigen that elicits neutralizing antibodies in experimental animal models and represents a critical target for recombinant vaccine development [16]. Recent studies have shown that LTB-conjugated scEDIII expressed yeast upon oral feeding elicited both humoral and mucosal immune responses in mice [19]. A tetravalent virus-like particle vaccine designed to display the EDIII domain was shown to induce multi-serotype neutralizing antibodies in mice and macaques, which confer protection against ADE [44]. Previous studies have also shown that the consensus EDIII expressed in tobacco plants induced a significant cellular immune response in the form of interferon-γ production and polyfunctional T cells in both the CD4+ and CD8+ compartments [45]. Based on these studies and considering the diversity between serotypes, the scEDIII construct was used in the current study and retained its antigenic characteristics despite being conjugated with the M cell targeting ligand Co1 and anchored to the yeast cell surface. Western blot analysis using anti-dengue mAb confirmed the specificity and predicted molecular weight of the recombinant construct.

For an effective oral vaccine, elicitation of a mucosal immune response is critical. The mucosal M cells are the major portal that take up luminal antigens and initiate antigen-specific immune responses [46]. These M cells regulate tolerogenicity of the mucosal environment conjugated with efficient presentation of the antigen to the lymphoid organ for processing. Oral vaccines exploit advantages of M cells via M cell-targeting ligands that have the potential to deliver ligand-conjugated antigens into mucosal lymphoid organs and evoke conjugated antigen-specific systemic and mucosal immune responses [47]. The M cell targeting ligand Co1A conjugated to the C-terminus of EDIII was reported to enhance EDIII-specific immune responses in systemic and mucosal compartments by T-cell stimulation [24]. The current study includes M cell targeting ligand Co1 conjugated to the target antigen scEDIII to achieve efficient mucosal targeting. Its precise conjugation and expression was explored through western and northern analyses.

The recombinant construct Co1-scEDIII-AGA has been successfully expressed and anchored to the yeast cell surface, as validated through FITC fluorescent labeling using anti-Dengue mAb. Yeast surface display, the platform currently being used to incorporate recombinant proteins into the cell wall, has achieved significant success with countless applications in biotechnology, including bioconversion, edible vaccines, bioremediation, and bioseparation [43, 48–50]. In addition to the convenience of production, which lacks expensive and tiresome purification processes, yeast cell wall components known to possess natural adjuvant activity act synergistically as a delivery vehicle and an adjuvant enabling the recombinant proteins to be more immunogenic [51]. Furthermore, antigens expressed and displayed on the yeast cell surface possess dual advantages when used as oral vaccines, which includes easy access to antibodies and the target antigen being presented on a large surface in the vicinity of mucosal tissue for improved adsorption [42, 43]. The ability to resist thermal and chemical denaturation and proteolytic degradation in oral vaccines is essential. Surface display of the target protein increases structural stability, inducing thermal stability of the target protein [52]. Thus, *S*. *cerevisiae* expressing recombinant antigenic determinants on its surface was considered a good oral vaccine candidate.

Recombinant protein Co1-scEDIII-AGA being successfully displayed on the yeast cell surface and retaining its functional properties led us to further investigate its immunogenicity to characterize it as a vaccine candidate. We found that surface displayed Co1-scEDIII-AGA developed anti-dengue IgG titers at 18 days after the last immunization, showing elicitation of a humoral response that differed significantly from controls, including Mock and non-surface displayed scEDIII-Co1. Although, M cell targeting ligand Co1A conjugated only to the C-terminus of EDIII was previously reported to enhance EDIII-specific immune responses in systemic and mucosal compartments in *E.coli* expression system [24], it seemed that such effect was not relevant in yeast expression system and elicitation of immune response depended mostly on the surface display strategy. This continued to increase until 32 days post last immunization. Several instances of yeast surface displayed recombinant vaccines eliciting significant humoral and cellular immune response have been reported. A study using a neutralizing epitope fragment of ApxIIA toxin (ApxIIA#5) of the Korean *A*. *pleuropneumoniae* serotype 2 strain displayed on the yeast cell surface [34] revealed elicitation of significant humoral immune responses. H5N1 hemagglutinin surface-presented yeast triggered both humoral and cell-mediated immunity in mice [51]. Invoking a mucosal immune response through antigen-specific sIgA production is one of the prerequisites of a successful oral vaccine [52]. Fecal sIgA levels were elevated slightly in surface displayed Co1-scEDIII-AGA fed mice, around 25 days post last oral dose, reflecting stimulation by exposure of PPs to the antigen Co1-scEDIII-AGA. In the mucosal immune system, gut-associated lymphoid tissue, including PPs, play an important role in the induction of antigen-specific immune responses in the gut. Although the sIgA titers were low, upon further analysis, the numbers of AFCs in the lymphocytes isolated from the spleen and PPs differed considerably between Co1-scEDIII-AGA-administered mice and the Mock control, as well as the scEDIII-Co1 administered counterparts. Interestingly, this study showed that the IgG and sIgA titers increased significantly upon booster dose immunization of purified *E*. *coli*-expressed scEDIII antigen. This was in correlation with our earlier observation that a single intraperitoneal injection of purified scEDIII induced a rapid surge in an immune response, especially in WC-fed mice [19].

Although the immune responses in mice fed with WC surface displayed EDIII yeast were significantly higher than those fed with Mock yeast or intracellularly expressed EDIII yeast during the vaccination period, there were still high variations among mice in this group (Fig. 5). In our previous study [19], high variations in immune responses (IgG and IgA) were also observed in the mouse group fed with whole yeast cells expressing LTB-scEDIII. In spite of the advantage of protecting the antigen from gastric degradation, feeding whole cells inadvertently reduces the amount of the antigen available for uptake in Peyer’s patches. Based on these consistent results, we believe that the variations are ultimately related to differential uptake of the antigen in different mice.

Limitations of most oral vaccines are associated with problems linked to antigen breakdown in the harsh gastric environment, as well as to the highly tolerogenic gut environment. The relative elicitation of both humoral and mucosal immune responses confirmed the suppression of oral tolerance. This was in correlation with earlier reports of efficient antigen delivery into PPs through oral administration of M cell targeting ligand Co1-conjugated EDIII antigens [24].

Due to the presence of genetically distinct complex serotypes, an ideal dengue vaccine should induce a life-long balance and lasting immunity against all four DENV serotypes. Despite some limitations of scEDIIII as a potential dengue vaccine candidate [53], yeast-expressed scEDIII induces specific antibodies against EDIIIs from all four serotypes [54] and, together with its small size, scEDIII is an attractive tetravalent dengue vaccine model for dengue vaccine development. Our previous study involving the scEDIII gene, also applied in the current study, showed that recombinant scEDIII produced neutralizing antibodies upon oral immunization [19]. Thus, the current vaccine candidate, surface displayed Co1-scEDIII-AGA, represents an efficient dengue vaccine candidate, possessing neutralization capability towards all four serotypes.

With the most affected regions being resource limited countries, an effective vaccine must be economical to meet the demands of mass immunization. Thus, oral vaccines are particularly attractive [52, 55]. The current study presents a cost-effective, efficacious oral vaccination strategy for the administration of *S*. *cerevisiae*-surface displayed Co1-scEDIII-AGA. Without using complicated processing techniques, the described construct not only elicits both systemic and mucosal immune responses, but the antibodies produced due to its oral immunization are believed to possess neutralizing ability against all dengue virus serotypes.

## Conclusions

This study represents an enhancement to our previous attempt to design economical and effective dengue vaccine candidates. Oral administration of whole yeast cells engineered to surface display the targeting ligand fused synthetic antigen Co1-scEDIII-AGA produced a stronger immune response compared to non-displayed Co1-scEDIII. In addition, Co1-scEDIII-AGA fed mice further elicited a significant immune response, both humoral as well as mucosal, upon intraperitoneal booster dose. These findings suggest that the yeast surface displayed preparations of Co1-scEDIII-AGA recombinant protein administered in the form of whole yeast cells show promise as a potent oral vaccine candidate against dengue virus infection.
